# Diversity of Pummelos (*Citrus maxima* (Burm.) Merr.) and Grapefruits (*Citrus x aurantium* var. *paradisi*) Inferred by Genetic Markers, Essential Oils Composition, and Phenotypical Fruit Traits

**DOI:** 10.3390/plants14121824

**Published:** 2025-06-13

**Authors:** François Luro, Elodie Marchi, Gilles Costantino, Mathieu Paoli, Félix Tomi

**Affiliations:** 1 UMR AGAP Institut, Université Montpellier, CIRAD, INRAE, Institut Agro, 20230 San Giuliano, France; elodie.marchi@inrae.fr (E.M.); gilles.costantino@inrae.fr (G.C.); 2 UMR SPE 6134—Université de Corse—CNRS, Equipe Chimie et Biomasse, 20000 Ajaccio, France; paoli_m@univ-corse.fr (M.P.); tomi_f@univ-corse.fr (F.T.)

**Keywords:** SSR, indels, SNP, genetic origin, terpenoid compounds, GC-MS, genetic distance, polyembryony, pulp color, acidity

## Abstract

Pummelo (*Citrus maxima*) is an ancestral species that has given rise to several major citrus varieties, such as sweet orange (*C. x aurantium* var. *sinensis*) and grapefruit (*C. x aurantium* var. *paradisi*). This species is also cultivated and its fruit consumed, particularly in Asia. Over the course of evolution, the allogamous reproduction of pummelos and the absence of asexual multiplication have contributed to its diversification. To assess its phenotypic diversity and the chemical composition of leaf and peel essential oils, genetic analysis using DNA markers is an essential prerequisite to ensure the identity and if varieties belong to this species. Fifty-eight accessions classified as grapefruits or pummelos were analyzed using 42 SSRs, 4 Indels, and 36 SNP markers. Based on the allelic composition of these markers, 20 cultivars were detected belonging to pummelos, 18 cultivars to grapefruits, and 11 were interspecific hybrids. The grapefruit inter-cultivar SSR diversity is null. The genetic origin of five interspecific hybrids is elucidated. The level of phenotypic diversity and of essential oil composition corroborate the modes of diversification, with high levels for those resulting from crosses and very low levels for the group of grapefruit mutants. Only the characteristics of breeding selection (pulp color, acidity and aspermia) are variable in grapefruits. In the composition of leaf essential oils (LEOs), nine profiles were detected in grapefruits based on variations in six compounds (neral, geranial, β-phellandrene, γ-terpinene, (E)-β-ocimene, and β-pinene). The seven interspecific hybrids involving pummelo as one parent show particular LEO profiles but without specific compounds, with the exception of p-cymenene which is present only in Wheeny. The diversity of peel essential oils in pummelos is lower, but variations in γ-terpinene, β-pinene, limonene, and myrcene make it possible to define seven profiles. With genetic verification the chemical and phenotypic diversity of the two species, pummelo and grapefruit, revealed in this study can be used as a reference for behavior in a specific environment.

## 1. Introduction

Pummelos are a very ancient citrus species (*Citrus maxima* Burm. Merr.) originating from Southeast Asia [1], emerging from a speciation phase during the Miocene era (between 8 and 6 million years ago) [2]. They are also considered as ancestral species because they are parents of cultivated modern citrus taxa which are created by crossing them with other ancestral species such as mandarin (*C. reticulata* Blanco) to generate sour orange (*C. x aurantium* var. *aurantium*) and sweet orange (*C. x aurantium* var. *sinensis*), or grapefruit (*C. x aurantium* var. *paradisi*) [3]. These interspecific hybrids are called secondary species, and they have evolved mainly through mutations from the initial intergeneric hybrid tree. One of the most famous examples of a mutation related trait is present in bloody sweet oranges, where the pulp color is the consequence of an insertion of a transposable element in the promotor sequence of *Ruby* gene [4]. Only mutations affecting a phenotypic trait have been selected by growers or breeders and vegetatively propagated by grafting or cutting. Prior to human selection, the survival of these hybrids over time depended on apomixis, which in citrus fruit manifests itself in the development of somatic embryos from a maternal origin tissue called the nucellus [5]. This apomictic multiplication of secondary species related to pummelos is inherited from mandarin trees (*C. reticulata* Blanco) and is caused by the insertion of a MITE (a miniature inverted repeat of transposable element) near the CitRWP gene [6,7]. Unlike in mandarins, this mutation did not occur in pummelos. As with other ancestral species, sexual reproduction is the main mechanism for varietal diversification in pummelos. A gametophytic self-incompatibility prevents self-pollination in pummelos and only inter-varietal crosses diversified this species [8]. The pummelos allogamy reproduction is not present in other ancestral species, nor in sweet orange, bitter orange, and grapefruit. However, some mandarin varieties, such as Fortune, Ellendale, and clementines, inherited the self-incompatibility from pummelos and produce aspermic fruits only in isolated monoclonal orchards. Studies of genome sequences show that the chromosomes of many mandarin varieties still contain introgression of the pummelo genome from ancient interspecific crosses [2,9,10]. The maintenance of these pummelo genome introgressions throughout mandarin evolution suggests that they provide selective adaptive advantages during hybrid genotyping.

Evolutionary genetics studies have shown that these contrasting modes of reproduction between ancestral and secondary species have modeled the genetic diversity of each [11,12]. Using co-dominant genetic markers (isozymes, SNPs, SSRs), pummelos are characterized by high allelic and varietal diversity and the species is in panmictic equilibrium, whereas grapefruits have low allelic diversity, a lack of inter-cultivar polymorphism, and very high heterozygosity. Other genetic diversity studies corroborate these results [13,14]. The genetic diversity (SSR markers) and morphological diversity of Indian pummelo cultivars were high [15]. The same finding was obtained with pummelo varieties from Bangladesh [16]. Uzun et al. (2010) used ISSR markers to reveal polymorphism between 29 grapefruit and 5 pummelo cultivars and observed greater diversity within pummelos due to their diversification by sexual crosses [17]. Wang et al. (2019) achieved the same results with over 70 pummelo varieties from China, but also reported the absence of SSR marker polymorphism between mutant forms of the Guanxi Miyou cultivar [14].

Because of its geographical origins, the pummelo fruit is very popular in Asia, while the grapefruit, born in the Caribbean islands, is mainly grown in Florida, South Africa, and Mediterranean countries. Thanks to pummelo allogamy and parthenocarpy a single-variety orchard produces seedless fruit. Aspermia, also sought-after in grapefruit, has been acquired by different methods, such as (i) the selection for sterility resulting from natural or irradiation-induced mutations, such as the Star Ruby cultivar [18]; and (ii) the obtention of triploid hybrids which greatly reduce fertility [19]. The only commercially successful triploid hybrid is Oroblanco (or Sweetie), which results from a cross between a Siamese acidless pummelo and a tetraploid grapefruit [20]. Pummelos with pink or red flesh resulted from spontaneous mutations detected in orchards in white-fleshed cultivars.

The chemical composition of citrus volatiles has been intensively investigated and found to be mainly composed of mono- and sesquiterpenes [21]. Pummelo leaf essential oils (LEOs) often vary in composition according to variety. The proportion of major compounds are highly variable, with β-pinene (10 to 46%), (E)-β-ocimene (5 to 31%), limonene (3 to 19%), and neral (less 1% to 15%) [22]. The grapefruit LEOs composition can be roughly described as sabinene (50%), (E)-β-ocimene (10%), linalool (8%), and β-pinene (3%). Nevertheless, some studies also mentioned the punctual presence of γ-terpinene (up to 56.1%), β-pinene (up to 30.9%), and p-cymene (up to 12.5%) [23].

The composition of pummelo PEO varies drastically. Some authors describe a large dominance of limonene (81 to 96%) [24,25,26,27] while others observe lesser relative quantities of 46–54% [28], 15–48% [29], or 64% [30]. The composition of grapefruit PEO is less controversial as it is largely dominated by limonene, which is from 86% to 98% of total compounds [27,31].

The aim of this current study is to assess pummelo and grapefruit diversity through different prisms. These are at the genetic level, using SSR and SNP markers to verify taxonomic status (species membership) and assess inter-varietal relationships, and at the phenotypic level, including the morpho-physiological diversity of the fruit and chemical diversity (composition of leaf and peel essential oils).

## 2. Results

### 2.1. Genetic Diversity Between Grapefruit and Pummelo Accessions

The allelic composition of the 46 genetic markers revealed probable synonyms, i.e., the same genetic profile shared by several varieties such as Eilat and Eingedi pummelo, Bali pummelo and Gold grapefruit, Oroblanco and Sweetie, Jackson and Triumph, and 18 grapefruit cultivars (Table S1). The following six varieties classified in the grapefruit group have unique genotypes: Asahikan, Alanoek, Royal, Yama, Jackson (or Triumph), and Wheeny. Six SSR markers detect three alleles per locus for Oroblanco or Sweetie. This result is consistent with the triploid origin of these two varieties since they are derived from a cross between a tetraploid grapefruit and a diploid pummelo. The proportion of heterozygous loci in pummelos is much lower than in grapefruits. However, in Cuban, Hog, and Menara varieties the proportion is much higher than in other varieties. Wheeny and Asahikan are distinguished by unique alleles which are not present in the reference species, with three and nine specific alleles, respectively.

**Figure 1:**
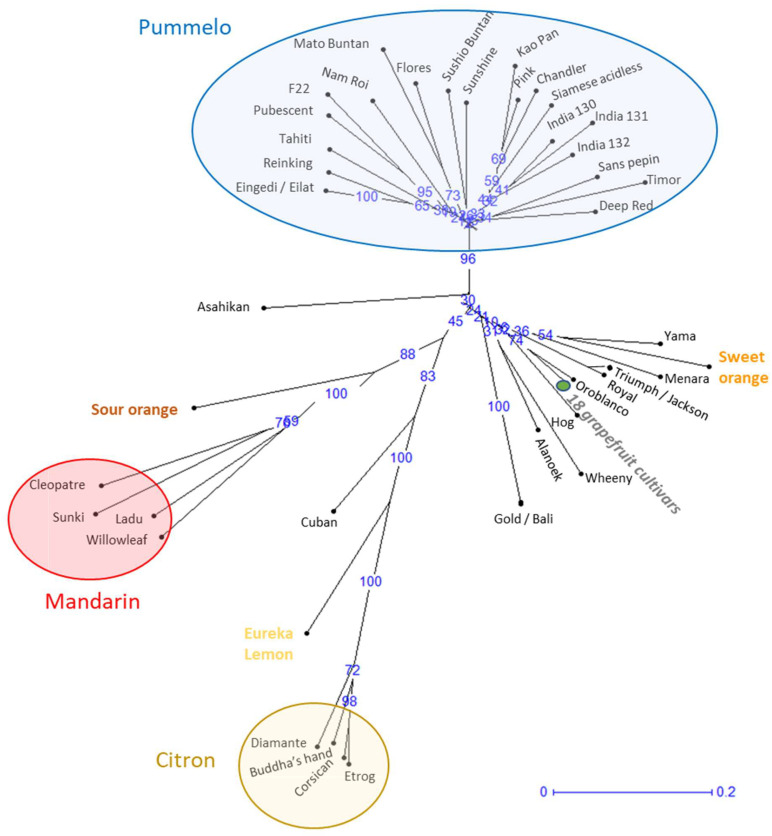
NJ tree of genetic relationships between pummelo and grapefruit accessions and representatives of the major *Citrus* species (Mandarin, citron, lemon, sour orange, and sweet orange) based on allelic data of 46 Indels and SRR markers. The blue numbers on the branches of the NJ tree represent the bootstrap values and the blue line indicates the branch length corresponding to a genetic distance of 0.2. The Marsh cultivar represents 18 other grapefruit cultivars.

Analysis of the genetic relationships based on genetic distances (Figure 1) highlights three main clusters corresponding to the three ancestral species (mandarin, pummelo, and citron) grouping varieties with small genetic distances between them. The pummelo cluster comprises 20 genotypes or 21 varieties, since Eilat and Eingedi are synonyms of the same genotype. The two genotypes of orange and grapefruit are localized in the same sector of the genetic diversity tree in agreement with their phylogenetic relation. Eleven other genotypes are distributed between these taxonomic groups. Of these, Oroblanco (or Sweetie) is the result of a breeding program involving a pummelo and a grapefruit variety. The 10 other genotypes have unknown origins and, contrary to their initial classification, can be considered as neither pummelo nor grapefruit. These 10 genotypes are therefore considered as non-true-type (NTT) varieties. The NTT genotypes corresponding to Yama, Menara, Triumph, Royal, Hog, Wheeny, Alanoek, and Gold are linked to the orange and grapefruit common branch, while Cuban is linked to the lemon and citron branch and Asahikan to the pummelo branch (Figure 1).

### 2.2. Genetic Origin and Taxonomic Position of Non True-Type Varieties

To elucidate the genetic origin of the NTT varieties, which differ from pummelo and grapefruit, and to verify their taxonomic position, we determined the origin of the alleles at each locus, defining their presence in each species. Only the most probable species were selected from the dendrogram of phylogenetic relationships to study the unknown origin of the nine genotypes (Table 1).

The proportion of loci carrying an allele of NTT varieties which are lower than 50% are not presented (Table 1). The proportion of sour orange never exceeds 67%, which excludes it from being a possible putative parent of genotypes of unknown origin. As with sour orange, grapefruit is probably not a parent of NTT varieties as the percentage of loci carrying a common allele never exceeds 95%. The totality of pummelo loci share a common allele with three genotypes (Triumph, Alanoek, and Wheeny), as do orange loci (Triumph, Royal, and Menara). Then, the Triumph (or Jackson) origin is the easiest situation to be resolved because only 100% of pummelo and 100% of sweet orange loci share at least one allele, and moreover their cross combination can reproduce the Triumph genotype in the 46 molecular marker loci. The origin of the other citrus hybrids is less obvious, as in no cross do we have all of the loci that match the genotype of an NTT citrus. A pummelo x sweet orange combination is the most probable cross that gave rise to Menara, Yama, Hog, and Royal as this assertion is supported by 90, 93, 95, and 98% of markers, respectively. A pummelo x citron cross is thought to be the origin of Cuban and this hypothesis is supported by 93% of markers. The origins of Alanoek and Bali are more uncertain as only 88% of markers support the best crossing hypotheses, which are pummelo × orange and pummelo × mandarin, respectively. The genetic origins of Asahikan and Wheeny remain unknown as no combination exceeds 71% of markers. These two cultivars are also the only ones with specific alleles (nine and three, respectively).

**Figure 2:**
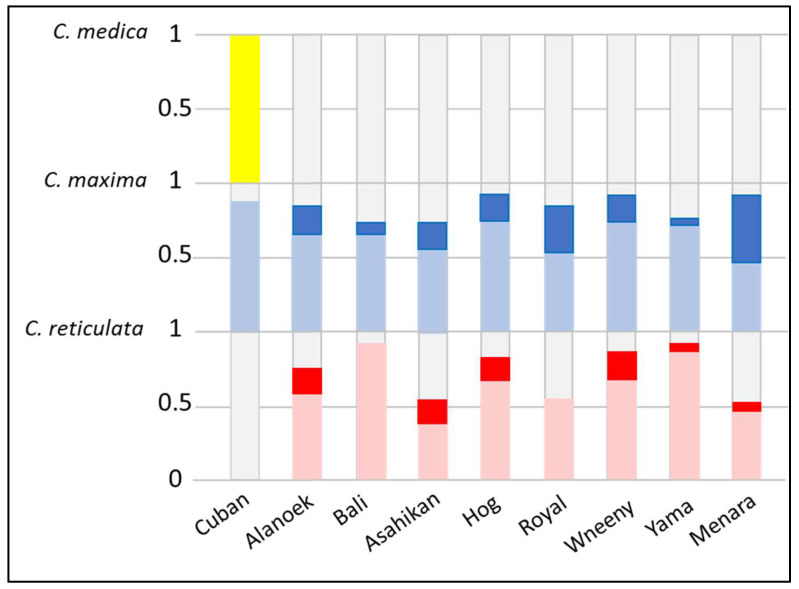
Stacked bar graph representing the proportion of ancestral species genomes of the NTT varieties and the frequency of homozygous and heterozygous SNP markers of the three basic taxa. Dark color for homozygous alleles; light color for heterozygous alleles; red/pink for *C. reticulata*; blue for *C. maxima*; and yellow for *C. medica*.

To verify the hypothesis of genetic origins and the identification of potential parental species, diagnostic SNP markers from three ancestral species, *C. medica*, *C. maxima*, and *C. reticulata*, were used to calculate the proportion of ancestral genomes and the frequency of homozygous and heterozygous alleles (Figure 2). The Cuban genome was constituted by 50% citron alleles, 45% pummelo alleles, and 5% citrus of unknown origin. The frequency of heterozygous markers of *C. reticulata* and *C. maxima* was high (100% and 90%, respectively). This profile confirms the interspecific hybrid status detected with the SSR markers. The frequency of pummelo’s homozygous markers for Menara and Royal (44% and 25%, respectively) conforms with the pummelo x sweet orange origin which was hypothesized by the SSR markers. With approximately 48% of *C. reticulata*, 38% of *C. maxima*, and 14% of citrus of unknown origin, and a very high proportion of heterozygous markers (95%), Bali and Yama are probably direct hybrids between mandarin and pummelo. The genomes of Alanoek, Hog, Wheeny, and Asahikan were constituted approximately by mandarin and pummelo alleles (plus a few undetermined alleles) and share a significant proportion (15–20%) of *C. reticulata* and *C. maxima* homozygous markers. Due to the low frequency of homozygous markers, those profiles are not compatible with a pummelo x sweet orange origin and probably belong to crosses between unknown interspecific (mandarin × pummelo) hybrids.

### 2.3. The Yield of Essential Oils

Cold pressed extractions were performed with the fruit peel of pummelos, grapefruits, and NTT varieties, resulting in essential oil yields ranging from 1.26% (Henderson) to 4.00% (Star Ruby), while grapefruit hybrid yields varied from 0.66% (Wheeny) to 7.25% (Oroblanco) (Table S2). As a synonym, Sweetie has a yield close to that of Oroblanco (6.99%). Extraction by hydrodistillation gave much lower EO yields than by scratching, on average 0.24% vs. 3.14%. However, Sweetie’s yield is still the highest. The yield of pummelo’s hydrodistillated LEO yields range from 0.29% (Chandler) to 1.29% (Eingedi) and are on average almost three times higher than those for other pummelo varieties (0.61%).

### 2.4. LEO Composition of Pummelo and Grapefruit

**Figure 3:**
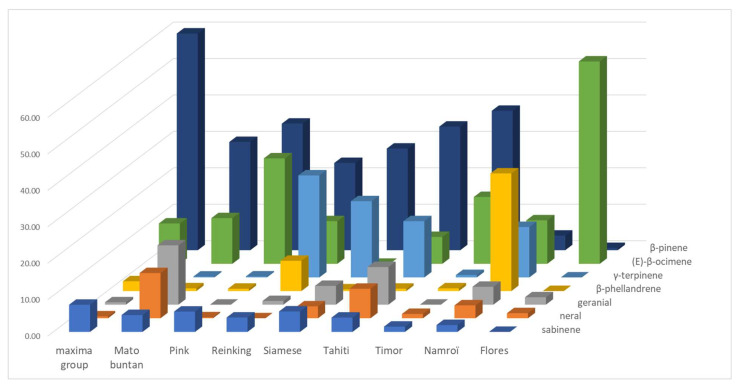
Relative quantity (%) of the 7 major LEO compounds detected in *C. maxima* varieties; the maxima group summarizes a mean profile of 11 cultivars with similar LEO composition (Chandler, Deep Red, Eilat, Eingedi, F22, Kao Pan, Sans Pépins, Sunshine, Indus G10, Indus G11, and Indus G13).

An LEO profile emerges from the batch which is shared by 11 pummelo cultivars (Chandler, Deep Red, Eilat, Eingedi, F22, Kao Pan, Sans Pepins, Sunshine, and Indus G10, G11, and G13), which are named the maxima group (Figure 3 and Table S3). This profile is made up of the following majority compounds: β-pinene (from 50.16 to 66.54%), (E)-β-ocimene (average 11.07% ± 3.72), and sabinene (average 7.47% ± 1.07). Among them, the three Indus cultivars stand out for a compound often absent from other cultivars, (E)-hex-2-enal (approx. 1.10%), but Indus G13 stands out from the other two cultivars for a high proportion of linalool (11.78%). Cultivar F22 stands out for its lower proportion of (E)-β-ocimene (3.60%) and higher of geranyl acetate (2.20%), while the other 11 pummelo cultivars have little or no geranyl acetate. The rest of the pummelo cultivars have much more heterogeneous profiles. Seven of them (Mato Buntan, Pink, Pubescent, Reinking, Siamese, Tahiti, and Timor) have a majority of β-pinene (average 32.00% ± 4.96), but differ in the second-most present compound. For Mato Buntan it is geranial (16.28%) followed by (E)-β-ocimene (12.54%) and neral (12.41%), for Pink and Timor it is (E)-β-ocimene (28.94% and 18.34%, respectively), and for Pubescent, Reinking, Siamese, and Tahiti it is γ-terpinene that comes second (20.20%, 28.00%, 20.92%, and 15.43%, respectively). For Siamese and Tahiti, geranial comes third (5.15% and 10.32%).

Finally, Nam Roi and Flores exhibit very different profiles from all the other pummelos. Nam Roi’s LEOs composition is characterized by β-phellandrene (32.28%), γ-terpinene (13.84%), and (E)-β-ocimene (11.93%) while Flores’ is largely dominated by (E)-β-ocimene (55.53%). The proportion of β-pinene is low in the LEOs from Nam Roi and Flores (4% and 0.61%), whereas it is over 23% in all other cultivars.

**Figure 4:**
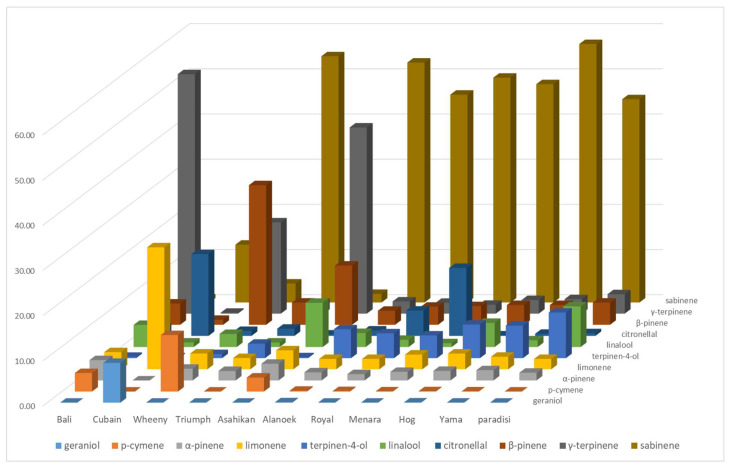
Relative quantity (%) of 10 major LEO compounds detected in grapefruit cultivars and in NTT varieties which are supposed to be pummelo’s hybrids (‘paradisi’ is a mean LEO profile of 18 true-grapefruit LEO profiles).

The LEO profiles of grapefruit cultivars are less variable, except for the four NTT varieties which are supposed to be pummelo’s hybrids which are very different (Yama, Wheeny, Gold, and Asahikan) (Figure 4). The basic LEOs profile of the 18 grapefruit cultivars is defined according to the following composition: sabinene (47.09% ± 5.60), (E)-β-ocimene (9.19% ± 2.24), terpinen-4-ol (8.46% ± 2.88), linalool (6.72% ± 3.73), and β-pinene (4.24% ± 0.64) (called ‘paradi’ in Figure 4). The LEOs profile varies with NTT varieties. The proportion of linalool is lower than ‘paradisi’ average for Triumph, Royal, Sweetie, and Oroblanco (maximum 1.60%). Sweetie has the highest proportion of sabinene (60.20%) and Oroblanco the highest for terpinen-4-ol (17.3%). Among the four singular profiles, Wheeny is characterized by β-pinene (30.90%), γ-terpinene (20.20%), and p-cymene (12.50%), whereas p-cymene (41.20%), β-pinene (13.10%, (E)-β-ocimene (9.90%), and linalool (9.80%) are the major components for Asahikan. Gold exhibited γ-terpinene as its major component (56.10%) and had a chemical composition very close to Bali. Cuban exhibits an original composition with an association of p-cymene (27.00%)/citronellal (18.10%) and a noticeable amounts of neryl/geranyl derivatives (nerol, neral, geraniol, geranyl acetate, and neryl acetate). Citronellal (18.10%) is also found at an appreciable amount in Menara, which is dominated by sabinene (49.76%).

**Figure 5:**
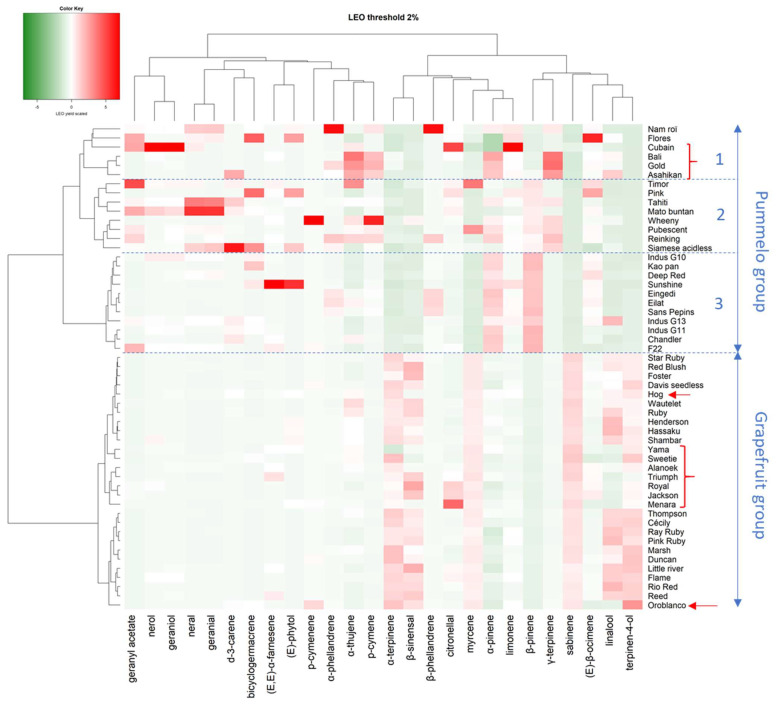
Heat map representing the diversity of pummelo and grapefruit cultivars according to their LEO composition only with compounds of which the relative concentration is higher than 2%. The red arrows and brackets indicate the position of the non-true-type varieties supposed to be pummelo’s hybrids.

Twenty-six compounds have relative quantities greater than 2% in at least one cultivar. The diversity of pummelo’s family, including grapefruit and pummelo’s hybrids, was analyzed with these compounds, whose proportions have been centered and reduced (Figure 5). The global diversity is structured into two main clusters, one grouping almost all grapefruit cultivars and the other all pummelo cultivars. This separation is only based on seven compounds, α-terpinene, β-sinensal, myrcene, α- and β-pinenes, sabinene, and terpinen-4-ol. The grapefruit cluster also includes pummelo’s hybrids such as Hog, Yama, Sweetie, Alanoek, Triumph, Royal, Jackson, Oroblanco, and Menara. The distances between cultivars are low and the inter-varietal diversity is mainly based on the variation of five compounds (α-terpinene, β-sinensal, citronellal, linalool, and terpinen-4-ol). The pummelo cluster is subdivided into three distinct subgroups (1, 2, and 3). Subgroup 1 was constituted by two pummelo cultivars (Nam Roi and Flores) and four of pummelo’s hybrids (Cuban, Bali, Gold, and Asahikan). The genetic distances between these cultivars are high except for the Bali and Gold. Subgroup 2 bring together eight pummelo cultivars (from Timor to Siamese acidless) with heterogeneous LEO profiles. Finally, Subgroup 3 comprises 12 pummelos (from Indus G10 to F22) with very close and homogeneous LEO compositions.

### 2.5. PEO Composition of Grapefruit and Pummelo

All grapefruit cultivars have very similar PEO profiles, with limonene (93.05% to 96.01%), myrcene (around 2%), and nootkatone (0.15 to 0.94%) in the majority (Supplemental S6) The only differences between cultivars are observed on a few minor compounds, such as (E)-β-ocimene (from 0 to 0.34%) which is absent in Pink Ruby, or decanal which is absent in Rio Red but is generally present in the others at around 0.25%. Among assimilated grapefruits, three cultivars have very similar profiles but are also distinct from Asahikan, Gold, and Wheeny grapefruits, with around 88.5% of limonene and over 5% of γ-terpinene. The only notable difference between these three citrus fruits is the absence of nootkatone in Asahikan. The profiles of Alanoek, Royal, and Yama are similar to those of grapefruit cultivars. Oroblanco and Sweetie have identical profiles but differ from other grapefruit in having a relatively high octanal content (around 0.40%), which is often absent in other cultivars.

**Figure 6:**
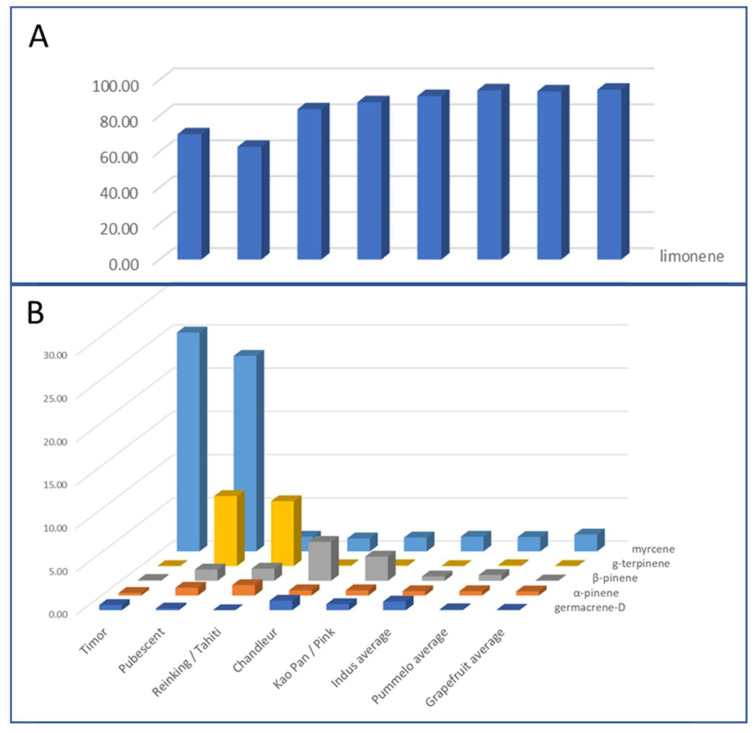
PEO profiles of the pummelo group described by the relative quantity (%) of 6 major aromatic compounds; (**A**) for limonene and (**B**) for the 5 compounds myrcene, γ-terpinene, β-pinene, α-pinene, and germacrene-D. The other means correspond to the average quantities between seven cultivars with a similar PEO profile (Deep Red, Eilat, Eingedi, Flores, F22, Sans Pepins, and Sunshine).

Pummelo’s PEO profiles are much more variable than grapefruit PEOs (Table S4). Seven compositions were distinguished based on 6 compounds (Figure 6). The LEO compositions of Kao Pan and Pink are similar, as well as Reinking and Tahiti and the three Indus cultivars. The last seven pummelo (Deep Red, Eilat, Eingedi, Flores, F22, Sans Pepins, and Sunshine) have compositions relatively similar to each other and an average profile has been composed with the average values of their aromatic compounds. Timor and Pubescent stand out from the other cultivars with a relatively low proportion of limonene (69.70% and 62.70%) and over 22% of myrcene. Reinking and Tahiti are characterized by limonene at only 83% and γ-terpinene between 7.50 and 8.40%. α-phellandrene is only present in the PEO of Eingedi, Eilat, Reinking, and Sans Pepin (between 0.30 and 0.50%). Chandler has the highest β-pinene content (4.50%), followed by Kao Pan and Pink with 2.80% and 2.20%. Eilat and Eingedi have identical profiles. Nootkatone is absent in the LEOs of five pummelo cultivars (Eingedi, Eilat, F22, and Reinking) and of one of pummelo’s hybrids (Bali). A higher proportion of germacrene-D was observed in Chandler and the three Indus cultivars.

Among pummelo assimilates the profiles of Bali, Alanoek, Gold, and Yama are close to those of Reinking and Tahiti, and the PEO of Hog and Menara is distinct from all other pummelo varieties due to the presence of decanal (0.2/0.3%). This compound is also found in the same proportions in grapefruit. The averages profiles of grapefruits and pummelos are close and they can be differentiated only by the proportion of β-pinene (0.04% ± 0.03 vs. 0.79% ± 0.37).

### 2.6. Phenotypic Diversity

The average values and standard deviations of the 13 fruit phenotypic descriptors were calculated (Table S5). From the mean values of the measured characteristics we can draw up typical phenotypic profiles or reveal major differences between grapefruits and pummelos (Table 2).

**Figure 7:**
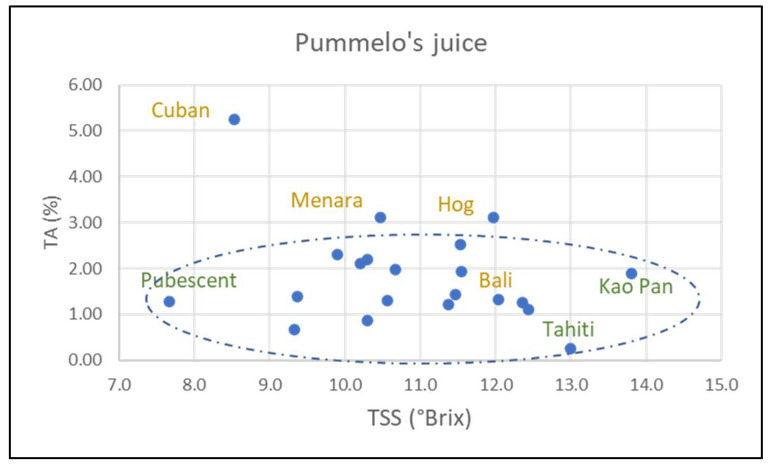
Dispersion of pummelo cultivars and hybrids according to the titratable acidity (% of citric acid) and total soluble sugar content (°Brix) (the pummelo cultivars are inside the circle); in green is the name of the pummelo, and in yellow is the name of the non-true-type varieties supposed to be pummelo hybrids.

In pummelo all characters are variable except for the number of embryos per seed, and to a lesser extent the pulp color (Figure S6a). Pummelo fruits are larger, with more segments, many more seeds, and all cultivars are monoembryonic. The most variable characteristic in grapefruit fruit is the pulp color (h) (Figure S6b). Fruit mass and number of seeds per fruit are also variable. All the characters are variable in pummelo’s hybrids (NTT varieties). However, these characteristics vary to a greater or lesser extent according to variety and species. Timor fruits are the heaviest (1453 g ± 321) of all pummelos, while Flores fruits are the lightest (490 g ± 60). Fruits from Sunshine, Sans Pepin, and Indus G13 are a flattened shape (0.64, 0.68, 0.70, respectively) while those from Eilat and Eingedi are oblong (1.17, 1.19). Skin thickness varies between 12.3 mm (Flores) and 28.1 mm (Timor). The number of segments varies between 14.3 (Pubescent) and 21.4 (Sunshine). Soluble sugar content (TSS) and acidity (TA) are the two most variable characteristics. The former ranges from 7.7 °Brix (Pubescent) to 13.8 °Brix (Kao Pan) and the latter from 0.25% (Tahiti) to 2.21% citric acid (Sans Pépin) (Figure 7).

Although most cultivars have an average seed mass of around 300 mg, the following two deviate significantly: Sunshine (432 mg ± 27) and Kao Pan (142 mg ± 14). All pummelo cultivars are monoembryonic. Skin chromaticity (C*) ranges from 47.3 (Pubescent) to 70.6 (Sunshine), while pulp chromaticity varies from 19.7 (Tahiti) to 10.5 (Pubescent). Skin color hue angle (h) ranges from 91.5 (Kao Pan) to 109.5 (Reinking). Phenotypic trait values for Eilat and Eingedi are virtually identical.

**Figure 8:**
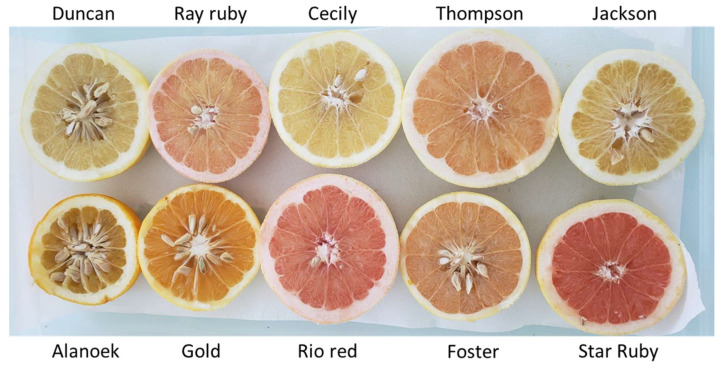
Photo of internal fruit pulp showing the range of color variation in the grapefruit group (Alanoek, Gold, and Jackson are non-true-type grapefruit but are pummelo’s hybrids).

Grapefruit weight and shape vary very little between cultivars, averaging 384 g ± 75 and 0.83 ± 0.02, respectively. Skin thickness varies between 7.5 mm (Star Ruby) and 12.87 mm (Little River). TSS and TS vary very little between cultivars. Duncan and Foster have an average of over 60 seeds per fruit, while the other varieties have less than 5. Pulp color varies widely, with yellow, orange, and pink colors (Figure 8). Star Ruby and Rio Red have the lowest hue angle and the highest chromaticity (C*), corresponding to a deep pink color while the other varieties have a pale yellow pulp.

There is greater phenotypic variability between NTT varieties (pummelo’s hybrid). There is greater phenotypic variability than in the other two groups. Among the most outstanding cultivars, Cuban stands out for its heavy fruit, very thick skin, low TSS, very high acidity (>5% citric acid), and monoembryony. Hog, Asahikan, and Menara also have a more acidic pulp (>3% citric acid) than the average grapefruit and pummelo. Sweetie has a flattened shape, a high number of segments (>15), and a low number of seeds per fruit. Cultivars with the highest TSS are Asahikan, Wheeny, and Gold (>12 °Brix). The phenotypic characteristics of Gold and Bali are identical.

**Figure 9:**
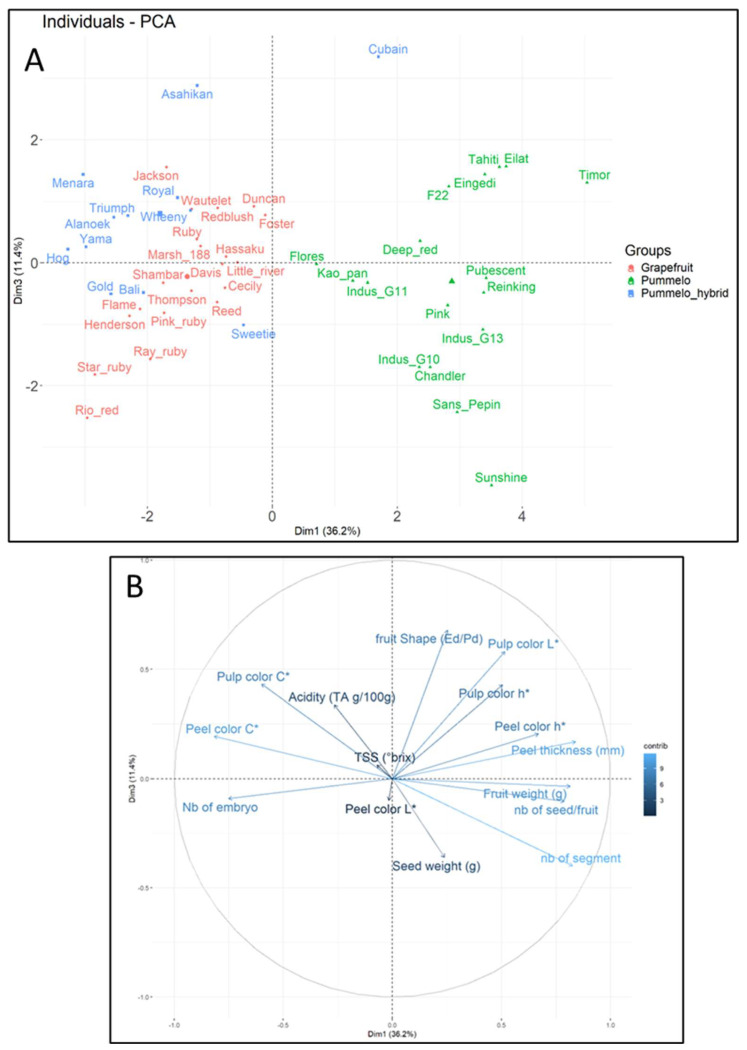
Phenotypic diversity of the pummelo family. (**A**) PCA representing the diversity of citrus varieties dispersed on the two first axis; (**B**) the contribution of phenotypic traits to the individual dispersion in the two first PCA axis.

Phenotypic diversity for all samples, collected using PCA, is shown to be structured (Figure 9). Despite their high intraspecific diversity, pummelo cultivars form a distinctly separate group from grapefruit cultivars and other hybrids. The central position of Kao, Pan, and Flores in PCA is essentially due to their lower seed mass than other pummelos. Cuban has a phenotype of its own, the distinguishing feature of which is the very high acidity of its pulp. Grapefruit cultivars are phenotypically similar, with only Star Ruby, Rio Red, Ray Ruby, Henderson, and Flame distinguished by their pulp color (h). Jackson’s phenotype is close to Royal’s. Sweetie’s phenotype is very similar to that of most grapefruit cultivars.

## 3. Discussion

### 3.1. Pummelo and Grapefruit Genetic Diversity

SSR markers were used to differentiate each ancestral species and so pummelo cultivars are grouped together in the same cluster because they share alleles not present in the other two ancestral species such as citron and mandarin. This interspecific allelic differentiation is linked to the allopatric evolution of ancestral citrus populations [2,10]. Nevertheless, intraspecific diversity is also high, because with the exception of the Israeli cultivars (Eilat and Eingedi) all other pummelo have a different genetic profile. This situation is a consequence of diversification through sexual crosses between varieties, which is similar for other ancestral species. In mandarins, interspecific crosses with pummelo occurred and were followed by back-crosses, with this contributing to introgressions of the pummelo genome into the genome of many mandarin cultivars [2,9,10]. Allogamy is another reproductive trait that has contributed to pummelo diversification by avoiding self-fertilization and achieving panmictic equilibrium [11]. This high varietal diversity revealed by SSR markers has already been highlighted in several studies [12,13,14,15,16].

The inter-cultivar genetic diversity of grapefruit is not in agreement with other studies [11]. In grapefruit, as in other secondary species such as sweet orange, sour orange, and lemon, varietal diversification has been based essentially on the selection of bud mutants, and reproduction has most often been vegetative thanks to apomixis (somatic embryogenesis) or horticultural clonal propagation techniques (grafting and cuttings) [11]. Consequently, there is no SSR polymorphism between cultivars of each of these taxa and a single cultivar is representative of the genotype (SSR profile) of the entire taxonomic group [12]. Mutations generate very little phenotypic diversity, except for traits that have been selected for improvement such as pulp color and number of seeds which are the only two truly variable traits.

Two rootstocks (Carrizo citrange or Pomeroy trifoliate orange) are used in our germplasm orchard. Can we suspect a rootstock effect on the variations observed between cultivars? Numerous studies have demonstrated that rootstock can modify scion fruit quality traits, such as made by Castle et al. (2010), where the performance of Valencia orange was studied on 12 genetically very different rootstocks [32]. The observed differences between Carrizo citrange and trifoliate orange were very small and often insignificant. The effect of rootstock on the composition of orange essential oils revealed only slight variations between citrange and trifoliate orange rootstocks [33]. Nevertheless, the secondary metabolite composition of a scion can be strongly modified according to the type of rootstock, especially in situations where the trees are under stress. Sour orange rootstock comparatively to Cleopatra mandarin, enhanced tolerance to spider mites of the scion by inducing flavonoids synthesis [34]. Among the grapefruit accessions in our study, three accessions are grafted on Carrizo citrange (Hassaku, Pink Ruby and Ray Ruby) and none showed significant differences from grapefruits grafted on trifoliate orange, irrespective of the traits studied. The aim of our study is not to evaluate precisely the minimal differences between cultivars, but to reveal a specific phenotypic profile in relation with the genetic divergence.

### 3.2. Pummelo and Grapefruit EO Diversity

The inter-varietal diversity of pummelos is also revealed by chemical and phenotypic analyses. Seven PEO and nine LEO profiles were detected among 20 pummelo cultivars and, if the leaf and peel EOs are combined then fourteen different aromatic profiles are obtained. Such variations in the quantities of the main LEO compounds had already been observed in eight Chinese varieties [20]. It is rare to find studies of essential oil compositions where the studied cultivars have previously been analyzed with genetic markers to attest to their taxonomic position. In the literature review on citrus EO composition it is mentioned that linalool does not exceed 0.1% and that γ-terpinene is often detected at between trace amounts and 0.05% [35]. In our study, we observed much higher proportions of these two compounds in some cultivars, for instance, linalool is at 11.78% in Indus G13 and γ-terpinene varies between 13.84% and 28.00% in five cultivars (Nam Roi, Reinking, Pubescent, Siamese acidless, and Tahiti).

Compounds presented as markers of pummelo’s PEO, such as trans-sabinene hydrate, γ-humulene, (Z,E)-α-farnesene, and δ-cadinene [36], are not always detected in the varieties in our study and only γ-humulene is present in five varieties and δ-cadinene is absent. However, these compounds are detected in the LEOs of nine pummelo varieties.

Citrus essential oils are mainly used in the cosmetics and perfume industries. Their composition determines their aromatic properties. It can be assumed that two EOs of identical composition will have very similar aromatic properties and can therefore be used interchangeably. Many factors can modify the EO composition, such as growing location, cultivars (genetic variability), ripening stages, storage conditions, or extraction methods [37]. Biotic and abiotic stress are also factors that induce the synthesis of new volatile compounds or modify the EO composition [38]. The terpenoids are a family of volatile organic compounds (VOCs) abundantly emitted by stressed plants [39,40]. Some of these compounds are involved in plant–environment interactions, either as pathogen inhibitors, chemical-endogenous signal-inducing abiotic-responsive genes or chemical messengers for plant-to-plant relation [41]. All the cultivars of our collection are healthy and under cultivating conditions avoiding any stress [42]. We can therefore conclude that the variations observed between cultivars are indeed in relation to their genetic constitution. On the other hand, the phenotypic peculiarities observed in our study for a group of cultivars or isolated varieties are the result of a combination of interactions between genetics and environment.

### 3.3. The Origin of Non-True-Type Varieties

The uncertainty of genetic origin is greater when certain markers have no alleles in common between the referent taxon and the genotypes studied, and especially when a secondary species is supposed to be the parent. This is the case for Alanoek, where 93% of markers suggest that orange could be one of its two parents. The representation of a species’ allelic diversity depends on its mode of diversification and reproduction, and the number of representative varieties. The representation of the allelic diversity of ancestral species then depends on the number of varieties chosen. The number of varieties representing *C. reticulata* and *C. medica* species is low in the current study (five for mandarins and four for citrons) and may therefore lead to an underestimation of the allelic diversity of these two species. SSR markers are not fully efficient markers for species diagnosis, which means that alleles are not present in all varieties of the species and that some may even carry alleles common to several ancestral species [43]. For these reasons, the frequency of markers with common alleles between ancestral species and NTT varieties is often less than 100%. On the other hand, when the frequency in secondary species is below 95%, their parentage may be called into question. Despite an equal frequency (88%), the cross-breeding hypothesis for the origin of Bali is more likely than that of Alanoek. In fact, due to the under-representation of ancestral species diversity, the probability of a pummelo x mandarin cross as the origin of Bali is still high. On the other hand, the pummelo x orange hypothesis at the origin of Alanoek is less likely as there is no diversity in SSR markers between orange varieties. So, a single variety represents the allelic diversity of the entire orange group. For two SSR markers, Alanoek and orange had no alleles in common. We can therefore conclude that one of Alanoek’s parents is a pummelo, but the other is not the orange, probably an unknown citrus variety with a genotype close to that of the orange. SNP markers diagnostic to ancestral species (DSNPs) have been developed for citrus and employed by Kaspar genotyping technology [44]. They have been used for the identification of genetic origin of pummelo/mandarin hybrids [9], of the Ichang lemon [45], or for the parents of limes and lemons [43]. Combined SSR and DSNP markers allow us to clarify the origin of Cuban, Menara, Royal, Bali, and Yama but not those of Alanoek, Hog, Wheeny, and Asahikan. The most likely hypothesis for these citrus is that they are the products of crosses between unknown pummelo/mandarin hybrids.

Some phenotypical and chemical traits indicated the hybrid origin of the outgroup varieties. For example the acidity of Cuban pulp is very high and could be supposed an inheritance of a citron parent [46]. In the LEOs the high proportion of p-cymene (27.00%), geraniol (8.80%), and nerol (5.90%) is also observed in citron LEOs [47]. The orange pulp color of Alanoek, Bali, and Gold is mainly related to the presence of β-carotene [48], suggesting a parentage within the mandarin group, including some mandarin hybrids such as sweet orange and sour orange. Unfortunately, the genetic data rules out the possibility that the orange and the sour orange are one of the parents. The polyembryony was also an indicator of the non-pummelo identity of these three varieties.

Wheeny’s LEOs profile stands out for the p-cymenene present at 2.30%, whereas it is mainly absent or sometimes below 0.40% in all the other cultivars in this study. In the literature, this compound is sometimes found in equivalent quantities in some mandarins such as satsumas [49], but not in sweet orange [50] nor in sour orange [51]. We can therefore assume that Wheeny is related to a mandarin or a mandarin hybrid, but not one included in our study. No compounds specific to the other unknown hybrids (Asahikan, Alanoek, and Gold/Bali) were detected, and their profiles are more or less a mixture of the grapefruit and pummelo profiles.

## 4. Material and Method

### 4.1. Citrus Accessions

This study was carried out on 25 pummelo cultivars (*C. maxima* (Burm.) Merr.) and 33 grapefruit (*C. x aurantium* var. *paradisi*) accessions (Table S6). The pummelo cultivars in this study have multiple Eastern and Western origins and can thus serve as a basis for comparison with studies carried out in different climatic conditions. All the cultivars are maintained in the INRAE-Cirad citrus germplasm (Iso9001 Biological Resource Center) at San Giuliano Corse, France (latitude 42°17′ N, longitude 9°32′ E). The trees were much more than 15 years old and grown in the same pedoclimatic and cultural conditions, with the exception of the rootstock, which is either Pomeroy trifoliate orange (*Poncirus trifoliata*) or Carrizo citrange (*C. sinensis* × *P. trifoliata*).

### 4.2. Genetic Analysis by SSR and Indel Genotyping

The required DNA is extracted from leaves. A piece of a leaf (about 100 mg) previously washed with ethanol is crushed with liquid nitrogen and mixed with 1 ml of extraction buffer preheated to 65 °C (Tris 0.1 M pH = 8; NaCl 1.4M, EDTA 20 mM, MTAB 2% wt/vol, PVP 1% wt/vol, sodium bisulfite 0.5% wt/vol). The resulting mixture is placed in a water bath at 65 °C for 20 min. To deproteinize the DNA and to solubilize the lipid an emulsion with an equivalent volume of chloroform–isoamyl alcohol (24/1, *v*/*v*) was added to the mixture. After centrifugation (10 min at 10,000× *g*) the supernatant was recovered in a new tube and mixed with 0.8 volume of isopropanol to precipitate the nucleic acids. The nucleic acids were recovered by centrifugation (5000× *g* 10 min). The pellet was washed with ethanol at 70%, dried, and then re-solubilized in ultra-pure water. The concentration was determined by the measurement of OD with a Nanodrop1000.

Genetic analysis was carried out using 42 SSR markers and 4 Indel markers (Table S7). All markers are distributed over the 9 chromosomes of the citrus genome [52]. PCR reactions were performed as simplex experiments in a 6 µL volume with 3 µL of PCR master mix (Qiagen kit), 0.35 µM of forward primer with a M13 tail at the 5′-end, 0.35 µM of reverse primer, 0.2 µL of fluorescently labeled M13-tail (6-FAM, NED, VIC or PET (Applied Biosystems, Foster City, CA, USA), 0.12 µL of 5 U/µL *Taq* DNA Polymerase (Taq’Ozyme OZYA001 from Ozyme, Montigny-le-Bretonneux, France), and 10 ng of DNA template. Amplified DNA samples were separated by a capillary electrophoresis-based 3130XL genetic analyzer (Applied Biosystems) with an internal standard. Data were analyzed with Genemapper™ software v5.0. The ADNid Company/Qualitech Group (Montpellier, France) performed genotyping.

### 4.3. Genetic Analysis by Diagmostic SNP Genotyping

For SNP genotyping with mandarin/pummelo diagnostic markers (DSNP), genomic DNA was extracted from the leaf samples using a DNeasy Plant Mini Kit (Qiagen S.A.) according to the manufacturer’s instructions. SNP genotyping was performed with a competitive, allele-specific dual Förster resonance energy transfer (FRET)-based assay. Detailed information about this genotyping method can be found in Curk et al. (2015) [44]. Thirty-six SNP markers were used with specific alleles of each of the three ancestral species, *C. reticulata*, *C. medica*, and *C. maxima* (18 SNP markers per ancestral species—2 per chromosome). Based on raw KasPar data, the heterozygous and homozygous allele frequencies of the three basic species were plotted on a split bar chart.

### 4.4. Phenotypic Characterization

The characterization of the fruits was carried out 10 months after their setting (in March). The aim is to describe the varieties on the basis of criteria related to the fruit.

For very seedy fruits (more than 5 seeds per segment) the counting of the totality was too tedious and so we listed the number of seeds in 3 segments, calculated an average of one segment, and multiplied it by the number of segments to obtain an estimated value per fruit. The shape of the fruit is described by the ratio of its diameter to its height, thus values lower than 0.9 translate to flattened fruits, close to 1 to round fruits, and higher than 1.1 of oblong or ovoid fruits.

The color of the fruit skin and pulp is estimated with a Chromameter CR 400 (Kanonica Minolta) thanks to three equidistant taps at the level of the equator of one fruit (reproduced with four fruits), against only one to determine that of the pulp. The color is determined by the three coordinates L, a*, and b*.

After mixing the juice of the 5 fruits of each tree, the acidity values of the fruit juice were obtained by titration with NaOH 0.1N using the apparatus (Mettler Toledo DL50 automatic titrator) and are expressed in milligram of citrate per 100 g of juice. The sugar content is expressed in Brix degree based on the angle of refraction of the light obtained with a refractometer (Hoeffer).

### 4.5. Peel and Leaf Essential Oils Extraction

For each cultivar, approximately 200 g of mature leaves and at least 10 ripe fruits were picked from locations on the same tree, early in the morning and in dry weather, from January to March 2022. The leaves were collected from three trees on the southeast-facing side and were located at the tree’s periphery. The peel of fresh fully ripe fruits was manually scaped with a zester and the essential oils were then separated from the zest crude extract by centrifugation (10 min at 8000× *g*). Fresh leaves were subjected to water distillation for 2 h 30 using a Clevenger-type apparatus in 2 L flask with a ratio of 200 g of leaves/1 L of distilled water. To avoid any damage, the oil samples were stored at −20 °C in amber vials until analyzed.

Peel essential oil (PEO) yields were calculated using the essential oil/fresh weight zest ratio. The yields of leaf essential oils (LEOs) were not calculated because the volumes of LEOs were too low to have a precise measure.

### 4.6. EO Analysis

The method, protocol, and apparatus for essential oil analysis are identical to the team’s previous publication [50]. Each sample was analyzed via dual column gas chromatography GC(FID) and gas chromatography combined with mass spectrometry (GC-MS) in order to determine the chemical composition. A few samples were also analyzed by ^13^C NMR following a methodology previously described [53].

#### 4.6.1. Gas Chromatography (GC) Analysis

GC analyses were performed on a Clarus 500 FID gas chromatograph (PerkinElmer, Courtaboeuf, France) equipped with two fused silica gel capillary columns (length 50 m, internal diameter 0.22 mm, and film thickness 0.25 μm), BP-1 (polydimethylsiloxane), and BP-20 (polyethylene glycol). The oven temperature was programmed to increase from 60 to 220 °C at 2 °C/min and then to hold in an isothermal state at 220 °C for 20 min. The injector temperature was 250 °C; FID temperature: 250 °C; carrier gas: hydrogen (1.0 mL/min); and the split was 1/60. The relative proportions of the oil constituents were expressed as percentages obtained by peak area normalization without using correcting factors. Retention indices (RIs) were determined relative to the retention times of a series of *n*-alkanes with linear interpolation (‘Target Compounds’ software of PerkinElmer, Courtaboeuf, France). The essential oil (EO) samples (≈30 mg) were diluted in 0.5 mL of chloroform.

#### 4.6.2. Mass Spectrometry Analysis

EOs were analyzed with a PerkinElmer TurboMass detector (quadrupole, Perkin Elmer, Courtaboeuf, France), coupled directly to a PerkinElmer Autosystem XL (PerkinElmer) equipped with a fused silica gel capillary column (length, 50 m; internal diameter, 0.22 mm; film thickness, 0.25 µm) and a BP-1 (polydimethylsiloxane). Helium was used as the carrier gas at 0.8 mL/min, 1/75 split injection, and 0.5 µL was injected. The injector temperature was 250 °C. The oven temperature was programmed to increase from 60 to 220 °C at 2 °C/min and then hold at an isothermal state for 20 min. The ion source temperature and energy ionization were set at 250 °C and 70 eV, respectively. Electron ionization mass spectra were acquired over a 40–400 Da mass range. Oil samples (≈30 mg) were diluted in 0.5 mL of chloroform.

#### 4.6.3. NMR Analysis

^13^C NMR analyses were performed on an AVANCE 400 Fourier-transform spectrometer (Bruker, Wissembourg, France) operating at 100.623 MHz for ^13^C, equipped with a 5 mm probe, in CDCl_3_, and with tetramethylsilane (TMS) used as internal reference. ^13^C NMR spectra were recorded with the following parameters: pulse width (PW): 4 µs (flip angle 45°); acquisition time: 2.73 s for 128 K data table with a spectral width (SW) of 220,000 Hz (220 ppm); CPD mode decoupling; and digital resolution 0.183 Hz/pt. The number of accumulated scans ranged from 2000 to 3000 per sample (≈30 mg of oil sample in 0.5 mL of CDCl_3_). Exponential line broadening multiplication (1.0 Hz) of the free induction decay was applied before Fourier-transform.

#### 4.6.4. Identification of Individual Components

The components were identified via three methods. The first one was a comparison of their GC retention indices (RIs) on polar and apolar columns, determined relative to the retention times of a series of *n*-alkanes with linear interpolation (‘Target Compounds’ software of PerkinElmer) to those of authentic compounds. The second one was based on computer matching against commercial mass spectral libraries and by comparison of spectra with literature data. The last method, used for few samples, was based on a comparison of the signals in the ^13^C NMR spectra of EOs with those of reference spectra compiled in the laboratory spectral library. In the investigated samples, NMR identified individual components at contents as low as 0.5% [53].

### 4.7. Data Analysis

Chemical data were analyzed using R v3.6.3 software (2020) with the g-plots packagev3.0.4 to analyze the EOs data and determine the relationships between cultivars and components contributing to this diversity. A heatmap clusterization was constructed with a function of the package FactoMineR v2.7.

The allelic data obtained with the SSR and Indel markers were used to calculate a genetic dissimilarity matrix using the simple matching dissimilarity index between pairs of accessions (units) on the Darwin v6 software [54]. Unweighted neighbor-joining (NJ) analyses were computed to describe the population diversity organization, and robustness of branches was tested using 1000 bootstraps.

## 5. Conclusions

Thanks to genetic markers it was possible to define taxonomic group membership or hybrid origin for all pummelo and grapefruit accessions under evaluation. For some of these outgroups, a hypothesis of cross-origin was proposed and discussed, with the support also of morpho-chemical descriptors. It seems that all outgroups originate from crosses involving pummelos, but none of them seem to have any link with grapefruits or sour oranges. Some synonyms have been revealed, such as Bali and Gold, Eingedi and Eilat, and Triumph and Jackson. This genetic analysis is an essential prerequisite for correctly assessing the phenotypic diversity of a species. Due to varietal diversification based on mutation, grapefruits show no SSR polymorphism and phenotypic variation limited to the selection criteria (seedlessness, color, and acidity). Pummelos (*C. maxima*), on the other hand, are highly diverse both chemically and phenotypically. The monoembryonic characteristic is present in all cultivars. In terms of leaf essential oils, seven profiles were detected out of twenty cultivars studied, with quantitative variations targeting six major compounds (neral, geranial, β-phellandrene, γ-terpinene, (E)-β-ocimene, and β-pinene). PEO diversity is lower due to the dominance of limonene, but variations were observed for monoterpene hydrocarbons such as γ-terpinene, β-pinene, and myrcene. These variations are consistent with allogamous reproduction in this species.

The results of a phenotypic characterization of genetic resources are the expectations of breeders as it enables them to choose the varieties they will be able to combine by sexual crossing or somatic hybridization according to their traits and characteristics. The monoembryonic trait, for example, is useful for selecting maternal parents as polyembryony is a handicap for obtaining offspring due to competition between embryos for germination and growth. The chemical composition of essential oils is also of interest in the search for new aromatic profiles, and for identifying molecules that can be used as identifiers of varieties or of the environment in which the trees were grown (tracers of geographical origin).

## Figures and Tables

**Table 1:** Investigation of genetic origin of pummelo’s hybrids by analyzing percentage of loci for each species sharing at least one allele of each outgroup variety. The number of specific alleles of outgroup varieties cross with a higher percentage of loci reproducing the genotypes of outgroup varieties (Pum: pummelo; grapef: grapefruit; mand: mandarin).

Species	Percentage (50%) of Loci Sharing at Least One Allele of Outgroup Variety									
	Triumph	Alanoek	Bali	Asahikan	Hog	Royal	Wheeny	Yama	Menara	Cuban
Pummelo	**100**	**100**	**93**	**88**	**98**	**98**	**100**	**95**	**93**	**95**
Mandarin	56	67	**95**	64	62	64	76	69	55	-
Orange	**100**	**93**	74	67	**98**	**100**	**83**	**98**	**100**	-
Sour orange	62	67	52	55	52	57	67	62	57	50
Grapefruit	95	86	79	**81**	88	95	**86**	74	86	76
Citron	-	-	-	-	-	-	-	-	-	93
Lemon	52	-	-	-	-	-	-	-	-	74
**Specific alleles**	0	0	0	**9**	0	0	**3**	0	0	0
Crosses	**Best % of loci reproducing the outgroup variety genotype for putative crosses**									
Pum × orange	**100**	**88**	-	-	**95**	**98**	71	**93**	**90**	-
Pum × grapef.		-	-	45	-	-	60	-	-	-
Pum × mand.		-	**88**	-	-	-	-	-	-	-
Pum × citron		-	-	-	-	-	-	-	-	**93**

**Table 2:** Phenotypic description of grapefruit, pummelo, and pummelo’s hybrid groups based on mean values and standard deviation (SD) of each parameter.

		Fruit Weight g	Fruit Shape (E/P)	Peel mm	Segment nb	TSS °Brix	TA % Citric Acid	Seed/Fruit nb	Seed Weight mg	Embryo/Seed nb	Peel Color		Pulp Color	
											C	h	C	h
**Pummelo**	**Mean**	**789.6**	**0.90**	**17.9**	**18.1**	**10.9**	**1.51**	**124.1**	**302.4**	**1.0**	**55.3**	**91.8**	**14.9**	**101.5**
	SD	259.0	0.15	4.7	2.1	1.5	0.62	31.9	67.9	0.0	5.3	2.8	2.1	6.1
**Pummelo hybrid**	**Mean**	**437.9**	**0.87**	**9.7**	**11.2**	**11.3**	**2.43**	**38.4**	**259.5**	**1.7**	**70.7**	**82.6**	**22.3**	**96.2**
	SD	288.8	0.08	5.6	1.4	1.1	1.14	16.1	66.5	0.7	6.5	6.4	4.5	6.1
**Grapefruit**	**Mean**	**380.4**	**0.84**	**9.6**	**13.1**	**10.2**	**1.84**	**9.49**	**258.4**	**2.5**	**66.9**	**88.0**	**17.6**	**90.3**
	SD	77.5	0.02	1.9	0.8	0.8	0.12	19.8	45.0	0.3	5.1	5.6	3.14	15.9

## Data Availability

Data are contained within the article and Supplementary Materials.

## Supplementary Materials

The following supporting information can be downloaded at: https://www.mdpi.com/article/10.3390/plants14121824/s1, Table S1: Allelic composition of SSR and Indel markers of the citrus accessions used in genetic diversity analysis; Table S2: Varietal yield of cold pressed and distillated PEO; Table S3: LEO compositions of; Table S4: PEO compositions of; Table S5: Fruit descriptor’s data; Table S6: List of citrus accessions; Table S7: SSR and Indel primers description; Figures S1: (a) Photos of pummelo fruits and (b) photos of grapefruit fruits.
