# Supplementary material for: Diversity of Pummelos (Citrus maxima (Burm.) Merr.) and Grapefruits (Citrus x aurantium var. paradisi) Inferred by Genetic Markers, Essential Oils Composition, and Phenotypical Fruit Traits
Source: Plants (Basel). 2025 Jun 13;14(12):1824. doi: 10.3390/plants14121824 (PMC12196667; doi:10.3390/plants14121824)
Supplement: Supplementary file 1 [file plants-14-01824-s001.zip › Supplementale file 8 Photos fruits maxima & paradisi.pdf]

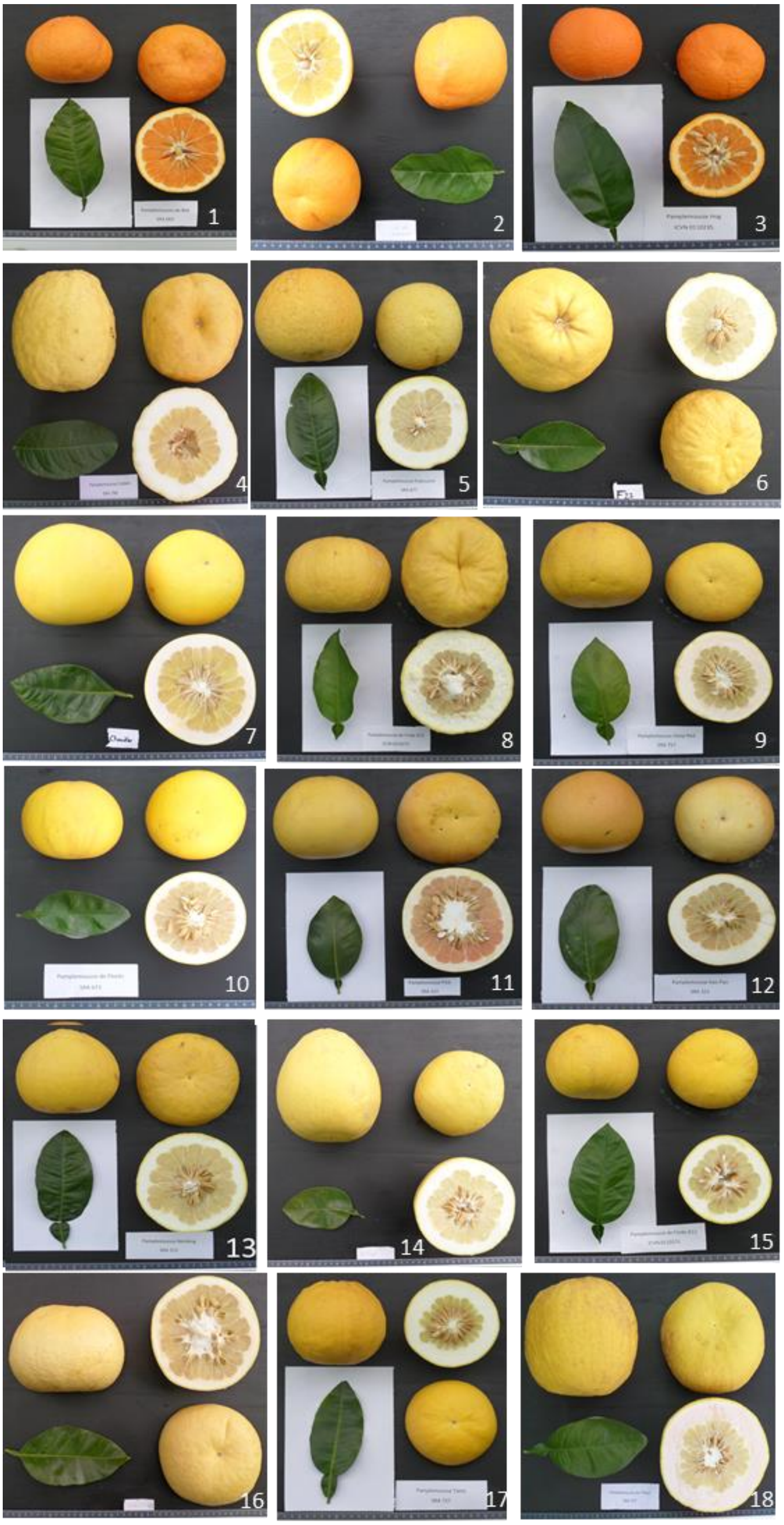

Figure SupFile 8 a : Fruit and leaf photos of 18 pummelo cultivars. 1: Bali, 2: Menara, 3: Hog, 4: Cuban, 5: Pubescent, 6: F22, 7 : Chandler, 8 : Indius G13, 9 : Deep Red, 10 : Flores, 11 : Pink, 12 : Kao Pan, 13 : Reinking , 14 : Eilat, 15 : Indus G11, 16: Sans Pépin, 17: Tahiti, 18: Timor

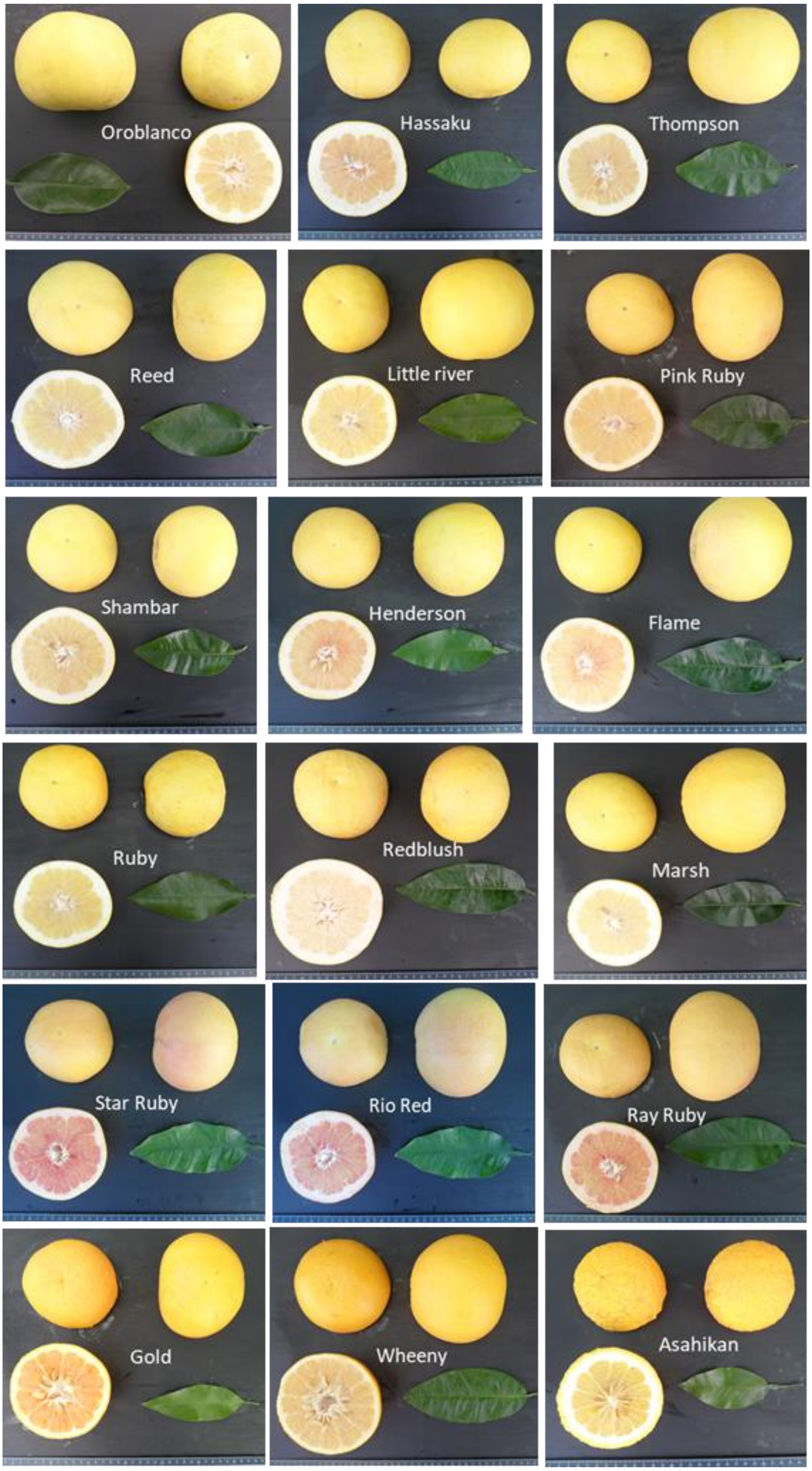

Figure Figure SupFile 8 b : Fruit and leaf photos of 18 grapefruit cultivars
